# Adapting and implementing breast cancer follow-up in primary care: protocol for a mixed methods hybrid type 1 effectiveness-implementation cluster randomized study

**DOI:** 10.1186/s12875-023-02186-3

**Published:** 2023-11-09

**Authors:** Sarah J. Fadem, Benjamin F. Crabtree, Denalee M. O’Malley, Lisa Mikesell, Jeanne M. Ferrante, Deborah L. Toppmeyer, Pamela A. Ohman-Strickland, Jennifer R. Hemler, Jenna Howard, Alicja Bator, Ayana April-Sanders, Rachel Kurtzman, Shawna V. Hudson

**Affiliations:** 1 Department of Family Medicine and Community Health, Research Division, Rutgers Robert Wood Johnson Medical School, New Brunswick, NJ USA; 2 Rutgers Cancer Institute of New Jersey, New Brunswick, NJ USA; 3 Institute for Health, Health Care Policy and Aging Research, Rutgers University, New Brunswick, NJ USA; 4 School of Communication and Information, Rutgers University, New Brunswick, NJ USA; 5 School of Public Health, Rutgers University, New Brunswick, NJ USA; 6 NORC at the University of Chicago, Bethesda, MD USA

**Keywords:** Cancer survivorship, Primary care, Breast cancer, EPIS, Practice change model, Implementation strategies

## Abstract

**Background:**

Advances in detection and treatment for breast cancer have led to an increase in the number of individuals managing significant late and long-term treatment effects. Primary care has a role in caring for patients with a history of cancer, yet there is little guidance on how to effectively implement survivorship care evidence into primary care delivery.

**Methods:**

This protocol describes a multi-phase, mixed methods, stakeholder-driven research process that prioritizes actionable, evidence-based primary care improvements to enhance breast cancer survivorship care by integrating implementation and primary care transformation frameworks: the Exploration, Preparation, Implementation, and Sustainment (EPIS) framework and the Practice Change Model (PCM). Informed by depth interviews and a four round Delphi panel with diverse stakeholders from primary care and oncology, we will implement and evaluate an iterative clinical intervention in a hybrid type 1 effectiveness-implementation cluster randomized design in twenty-six primary care practices. Multi-component implementation strategies will include facilitation, audit and feedback, and learning collaboratives. Ongoing data collection and analysis will be performed to optimize adoption of the intervention. The primary clinical outcome to test effectiveness is comprehensive breast cancer follow-up care. Implementation will be assessed using mixed methods to explore how organizational and contextual variables affect adoption, implementation, and early sustainability for provision of follow-up care, symptom, and risk management activities at six- and 12-months post implementation.

**Discussion:**

Study findings are poised to inform development of scalable, high impact intervention processes to enhance long-term follow-up care for patients with a history of breast cancer in primary care. If successful, next steps would include working with a national primary care practice-based research network to implement a national dissemination study. Actionable activities and processes identified could also be applied to development of organizational and care delivery interventions for follow-up care for other cancer sites.

**Trial registration:**

Registered with ClinicalTrials.gov on June 2, 2022: NCT05400941.

**Supplementary Information:**

The online version contains supplementary material available at 10.1186/s12875-023-02186-3.

## Background

Individuals living with a history of breast cancer are increasing in numbers and managing significant late- and long-term treatment effects and related symptom burden. Breast cancer is the most common cancer, excluding non-melanoma skin cancers, for women in the U.S. [1]. There are more than 3.5 million individuals with a history of invasive breast cancers in the United States (U.S.), with more than 2.6 million (75%) being 60 years of age or older [1]. Driven by innovations in early detection and adjuvant treatment, relative survival rates for breast cancer has continually improved [2], at 5-years (89%), 10-years (83%), and 15-years (78%) post-diagnosis resulting in surges in people living long-term after a breast cancer diagnosis [1]. Most of these individuals are diagnosed with localized breast cancers (61%), which have a relatively high survival rate (99%) [1]. Nevertheless, there are significant adverse late and long-term post-treatment effects. Adverse health outcomes post-treatment for breast cancer may include lymphedema [3], chronic pain [4–6], anxiety and depression [7, 8], sarcopenic obesity [9], bone loss and fracture risk [10, 11], declines in cognitive function [12–14], stroke [15], cardiovascular disease [16, 17], sexual dysfunction [18, 19], neuropathy [20, 21], and fatigue [22].

From 2012 to 2025, the overall market demand for oncology is projected to rise by 40% resulting in shortages of oncologists to meet both follow-up care and increasing treatment demand [23–25]. Adult cancer survivors acknowledge preferring follow-up care driven by cancer specialists rather than primary care [26]. Yet, the percentage of people with a cancer diagnosis visiting cancer and cancer-related physicians declines each year, and recent estimates suggest that their care continues in primary care settings [27]. Nearly 75% of women who had a history of breast cancer saw primary care clinicians, and these percentages did not decrease annually [28]. Serious challenges remain in transferring actionable information from cancer care to primary care, which is a significant problem for breast cancer populations who require long-term surveillance [26].

### Survivorship and primary care

There is unrealized potential for primary care to have a greater role in the care of patients with a history of breast cancer [29]. In two Canadian randomized controlled trials (RCTs), primary care clinicians were as effective at detecting recurrence in breast cancer survivors as hospital-based systems, with greater patient satisfaction [30] and no differences in psychosocial outcomes [31]. People with a history of breast cancer rate primary care clinicians higher for coordination of care and comprehensive care than oncologists for services that include tracking of care, ongoing management of medical problems and preventive care [32, 33]. However, the U.S. system is plagued with fragmentation between specialty and primary care prompting the American College of Surgeons’ Commission on Cancer (CoC) to initially mandate [34, 35] the implementation of survivorship care plans (SCP) as a communication tool [36, 37]. Yet, primary care guidelines and survivorship care plans have not produced the necessary changes for better care because they often do not address implementation challenges in the broader primary care context.

While SCPs have been shown to increase the likelihood that primary care clinicians report engaging in survivorship care planning [38], primary care practice innovators have voiced frustrations about the investments to support SCP development [36, 37] despite limited effectiveness in patient-reported outcomes [39, 40]. A third of patients with a history of cancer believe there is a shared care role for primary care in cancer follow-up, suggesting opportunities to perform routine cancer-screening tests, supplement cancer and cancer-related specialist care, and provide follow-up medical care when “enough time has passed” [26]. Yet, ‘shared care’ for patients with a history of breast cancer, where multiple teams (primary care, oncology, and medical subspecialists) jointly participate in care delivery, remains consistently understudied [41]. Studies investigating implementation activities to enhance primary care capacity to participate in shared care models are urgently needed [42, 43].

Cancer care delivery research is an emerging dissemination and implementation science research area and research priority for National Cancer Institute (NCI) [44]; however, many efforts to date have been insufficiently informed by implementation science theories and methods [45]. Most U.S. cancer survivorship studies focus on implementation of cancer survivorship care plans in oncology. Few address important context and process factors affecting their use in primary care or post-cancer treatment settings [40, 46, 47]. Few U.S. based studies explore implementation of evidence-based interventions (EBIs) for patients with a history of cancer [44], and similarly few studies address implementation of EBIs or scaling up in primary care settings [48]. A recent review of SCPs notes “survivorship care models in real-world settings will likely require moving beyond traditional randomized controlled trials to conduct research informed by implementation science methodology” [40]. Our study addresses these issues using a “designing for dissemination” perspective [49–51] while attending to important context, capacity and patient complexity factors impacting the implementation of breast cancer follow-up care in primary care.

### Conceptual frameworks

The design of this study integrates the Exploration, Preparation, Implementation, and Sustainment (EPIS) framework and the Practice Change Model (PCM). EPIS is a program implementation-based framework that provides assessment constructs for exploring inner and outer context factors that impact evidence-based intervention implementation [52–54]. Exploration is the act of evaluating whether the intervention fits the current environment. Preparation includes planning implementation and inventorying proposed challenges. Implementation focuses on the process of assuring and balancing fidelity to the evidence-based program (EBP) delivered with adaptations needed to assure program success. Sustainment focuses on maintenance and program and factors impacting implementation over the long haul. The PCM is based on complexity theory and was empirically derived through a mixed method comparative assessment of high and low performing primary care practices that implemented an intervention to improve delivery of services [55–57].

**Fig. 1:**
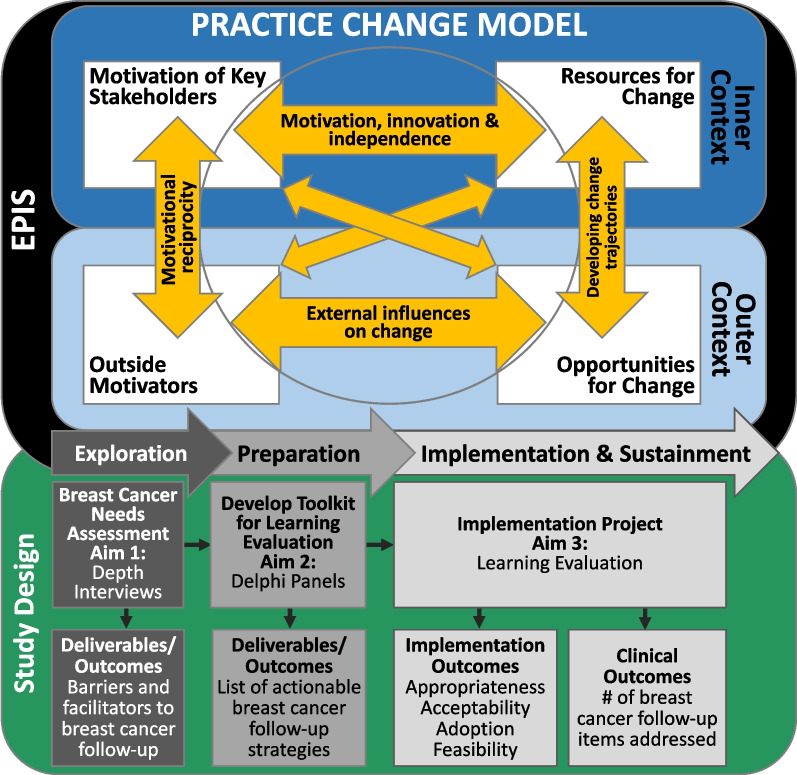
Conceptual model that blends the Exploration, Preparation, Implementation, and Sustainment (EPIS) framework and the Practice Change Model (PCM)

The PCM complements EPIS in important ways differentiating motivation (energy for change) and capacity or resources for change (capability). In addition, it emphasizes the importance of interdependencies that manifest among the contextual factors influencing intervention effectiveness and is used to guide the intervention. Figure 1 shows the relationship between the conceptual models being used (EPIS and PCM) and their associated role in the study design.

### Identifying actionable strategies: a multi-method stakeholder informed process

Early phases of this research were part of the Exploration and Preparation phases, while the current protocol describes the Implementation and Sustainment phases. The exploration and preparation phases used a combination of depth interviews and Delphi panels [58] to identify potential interventions. As part of the exploration phase, we executed a needs assessment through depth interviews with 40 national and local stakeholders representing patients/patient advocates, health care clinicians (separately primary care and oncology), policy influencers, and researchers to generate evidence from different perspectives on the role of primary care for patients with a history of breast cancer. Participants were recruited either via nomination by the grant advisory committee or through snowball sampling.

As part of the preparation phase, we used these findings to perform Delphi panels with 23 experts in care for patients with a history of breast cancer. Participants engaged in a 4-round online Delphi panel to identify strategies for defining and fostering primary care’s role in care for patients with a history of breast cancer. Innovators included primary care physicians, oncologists, researchers and policy influencers from government and professional organizations.

Guided by the insights from the exploration and preparation phases and PCM actionable evidence-based protocols for breast cancer survivorship care, we have created a list of actionable breast cancer follow-up strategies that will be implemented in a hybrid type 1 effectiveness-implementation cluster randomized study. The objectives of this intervention are to:

1. Determine priority recommendations for patients with a history of breast cancer that are most compatible for adoption in primary care practices.
2. Investigate the organizational and innovation adaptations needed to support the integration of priority recommendations for breast cancer follow-up care.
3. Understand how physicians and staff perceive the utility and sustainability of the priority recommendations for breast cancer patients in day-to-day practice operations.

## Methods

### Participants and procedures

This study will be conducted in 26 practices recruited from 79 primary care practices in the Robert Wood Johnson (RWJ) Barnabas Health system [59]. RWJ Barnabas Health includes 207 primary care clinicians serving 73,000 patients throughout New Jersey. Practices will be randomized in pairs, by approximate size and type of practice, into 13 intervention or 13 waitlist control. The impact of this implementation will be measured using mixed methods to assess EPIS Implementation and Sustainment factors related to how organizational and contextual variables affect adoption, implementation and early sustainability for provision of follow-up care, symptom, and risk management activities at 6- and 12-months post implementation. The methods of this study have been reported using the SPIRIT guidelines (Additional file 1).

### Intervention and implementation strategies

The goal of this intervention is to support primary care practices in adoption of actionable strategies aligned with priority recommendations of evidence-based activities (Table 1) for breast cancer survivorship care. To accomplish this, multi-component implementation strategies (Table 2) will be tailored to the unique needs of each clinic; these may include practice facilitation, [60–67] expert consultation, [68, 69] collaborative learning events, [60, 70–72] audit and feedback, [73, 74] and a learning evaluation [75]. Core components of this intervention include a learning collaborative among participating sites and a participatory engaged practice model in which practices are asked to determine what elements of survivorship care are most acceptable and feasible for their local context.

**Table 1:** ACS/ASCO Breast cancer evidence-based activities for practice customized implementation of survivorship care recommendations

Evidence-Based Symptom and Risk-Based Care Recommendations	Assess	Treat, screen or do^a^	Counsel/ provide education	Provide referral^a^
**Late and Long-term Effects**				
Fatigue	√	√		
Cognitive impairment	√	√		
Cardiotoxicity			√	
Distress/depression	√	√		
Pain and neuropathy	√	√		
Sexual health			√	√
Premature menopause	√	√		
**Lifestyle**				
Smoking cessation	√	√	√	
Obesity			√	
Physical activity			√	
Nutrition			√	
**Surveillance and Screening**				
Screening for recurrence	√	√	√	
History and physical	√	√		
De-implement MRI as low risk patients		√		
Genetic counseling for family history				√
Adjuvant endocrine therapy screening			√	
Cervical cancer screening		√		√
Colorectal cancer screening		√		

^a^Measurable in a searchable field in Electronic Health Record (EHR)

**Table 2:** Timeline for intervention activities & implementation assessment

Initial 13 Practices Waitlist Controls – 13 Practices	Year in months											
**Intervention Activities**	**1**	**2**	**3**	**4**	**5**	**6**	**7**	**8**	**9**	**10**	**11**	**12**
Learning collaborative	√											√
Practice facilitation	√	√	√									
Academic detailing/expert consultation	√	√	√	√	√	√						
Audit and feedback	√	√	√									
**Assessments**												
RWJ Barnabus BC EHR abstraction	√					√						√
Qualitative interviews with practice members and patients	√	√	√			√						√
Practice observation	√					√						√
Practice Staff Questionnaire	√					√						√

Practices will participate in a Learning Collaborative, beginning with a Kick-Off Meeting that will include opportunities for peer and traditional learning from expert faculty. A series of informational and motivational sessions will be led by members of our steering committee and project team. At least one physician and a project quality improvement (QI) champion from each practice will be invited to attend and work with the practice facilitator to develop a practice-specific intervention plan. We have also assembled an Implementation Advisory Committee (IAC) of internationally renowned health care opinion leaders and patient advocates engaged in cancer survivorship. The IAC will be convened throughout the study to provide guidance and feedback. At the initiation meeting, practices will be oriented to the rationale for implementation of evidence-based breast cancer care delivery in primary care. Practice members will then be asked to evaluate priority recommendations for appropriateness, feasibility, and acceptability for adoption within their specific setting. The intervention team will work with the practice leadership to reach consensus on an adoption plan and assess practice operations to identify adaptations and needed supports for implementation based on the adoption choice. This multi-faceted strategy will help participating practices incorporate both clinical and organizational recommendations and allows the research team to develop supports to ease implementation. We will use data in both a kick-off learning collaborative and later in individual practice facilitation to educate clinicians and staff members about their important role in care for patients with a history of cancer. A second, final learning collaborative will serve as a post-intervention debrief.

Practice facilitation by a trained practice facilitator is a keystone of our implementation strategy (PCM outside motivators) [60–67]. The practice facilitator will be trained in symptom and risk management activities. An initial step for each practice will be to create a registry of breast cancer patients in the practice. RWJ Barnabas Health monitors administrative data centrally and can pull this data, serving as a resource for this activity. The facilitator will work with the practices to assess workflows, train clinicians in the use of audit and feedback for breast cancer care metrics to identify gaps in care, and provide symptom and risk management activities. Audit and feedback will be used to share audit reports from registries created by the practices with support from the practice facilitator [76]. EHR and registry data will be used to trouble-shoot data discrepancies, discuss and identify improvement plans, and monitor improvement over time.

### Learning evaluation and data collection

We will use a learning evaluation strategy in which ongoing data collection and analysis are used to optimize adoption of the intervention [75, 77]. Qualitative observations and interviews will be performed to understand: (1) the overall practice environment in which the intervention occurs; (2) contextual features that enhance or inhibit adoption of the intervention; (3) any adaptations to the intervention to conform to contextual needs; and (4) how the intervention was implemented. Our assessment will be guided by the following research questions:

1. Which priority recommendations for breast cancer survivor care are most compatible for adoption in primary care practices? (Appropriateness and Adoption)
2. What organizational and innovation adaptations are needed to support the integration of priority recommendations for breast cancer survivor care? (Feasibility and Fidelity)
3. How do physicians and staff perceive the utility and suitability of the priority recommendations for breast cancer survivor care in day-to-day practice operations? (Acceptability)

To monitor implementation, a trained evaluation team member will conduct site visits at each intervention practice at baseline, 6 months, and 12 months. During these visits, we will observe work patterns and dynamics within each practice, how recommendations interface with daily workflows, how recommendations are introduced and explained to patients with a history of breast cancer, and how the recommendations affect coordination of cancer-related care with other chronic care needs. We will also observe how recommendations can be improved or optimized in each setting under current conditions. Fieldnotes will be prepared to record instances where contextual factors (e.g., physical space; organizational, clinician or patient features; care team workflows or processes) impact implementation efforts. Special attention will be paid to contextual features and interactions that were indicative of positive or negative alignments between intervention and local setting and instances where better alignments serve to enhance implementation progress (e.g., “implementation measures” as outlined in EPIS). Observational data will be captured in detailed fieldnotes and written up quarterly as case summaries for each practice and shared with our IAC for review and input.

During evaluation site visits, serial, open-ended key informant interviews with clinic members will be conducted. Interviewees will be purposefully selected based on observations during site visits that identify individuals who have the best insights on different aspects of the intervention. We seek to understand ongoing implementation issues and any contextual features that lead to both adoption and modifications to recommendations, and any impact of modifications on intervention effectiveness. These data are consistent with our conceptual framework (e.g., PCM “motivators/ resources for change” and EPIS “intervention characteristics”). Interview data relating to the fit of the intervention to the primary care context will then corroborated by observational fieldnotes taken during site visits. In addition, during the initial implementation and follow up assessment periods, we will conduct approximately 3–5 Zoom or telephone-based individual depth interviews per practice with patients with a history of breast cancer identified through medical records or by practice staff members [78, 79]. Interviews will focus on experiences of these patients in the practice and contextual features affecting their experience receiving cancer-related recommendations and referrals. This is consistent with EPIS “outer setting” and “intervention characteristics” and interrelationships between context and intervention as described by the PCM. Recorded interviews will use a semi-structured interview guide and be transcribed for analysis.

### Process data outcomes

During the initial meeting, we will ask practice members to assess the appropriateness, feasibility, and acceptability of the priority recommendations to inform an adoption decision. Feasibility will also be assessed based on the ability of practices to implement a functional breast cancer registry and the ability to use audit and feedback data to address patient symptom management needs. Usability will be assessed from usage tracking data collected from qualitative fieldnotes during the learning evaluation and acceptability from responses of staff to assessments (e.g., number and type of workflow changes). Adoption will be assessed through examination of practice members and organizational intervention use (e.g., number of activities implemented, number of referrals for symptom management).

### Practice level variables

Guided by EPIS, contextual factors being measured were selected based on suggestions from clinical stakeholders, community partners, and previous literature suggesting they may influence implementation success [79–81] (Table 3). Healthcare team background factors, implementation climate, and medical clinician background will be assessed using a Practice Information Form that collects demographics, management activities, and financial information for the practice and a Practice Staff Questionnaire (PSQ) [82]. The PSQ measures clinicians and office staff perceptions of key practice attributes such as “Relationship Infrastructure,” “Facilitative Leadership,” “Teamwork,” “Work Environment,” and “Culture of Learning” [83] chosen because the literature has identified them as key mechanisms for successful organizational change and patient care improvement [84, 85].

**Table 3:** Study assessments

	**Baseline, 6 mo. & 12 mo. post implementation**
**Practice Setting and Team-Level Variables**	
Implementation Climate	QUANT
Medical Provider Background (e.g., sex, race, years in practice)	QUANT
Facility Resources	QUAL
Organizational Readiness for Change	QUAL (+ quant)
Leadership Style	QUAL (+ quant)
Healthcare Team Communication Quality	QUAL
Patient-Provider Communication Quality (provider perceived)	QUAL
Demographics	QUANT
**Patient Background Variables**^**a**^	
Depression and Anxiety	QUANT
Cognitive Function	QUANT
Fatigue	QUANT
Pain	QUANT
Menopause	QUANT
Smoking status	QUANT
Obesity status (BMI)	QUANT
**Patient Primary Outcome**^**a**^	
# of BC follow-up items implemented	QUANT

^a^These assessments will be collected from the EHR for patients in all 26 practices during the initial and waitlist interventions to assess effectiveness. They will also be monitored at 18 and 24 months for initial intervention practices to assess sustainability

Facility resources and clinic spaces will be assessed using an observational quantitative checklist of space and resources at baseline and note any changes over the course of implementation. Qualitative interviews will address perceptions of facilities such as assets and deficits, satisfaction with facilities, and impact of facility on breast cancer follow-up care implementation.

Qualitative interviews will probe stakeholder perceptions of change in their healthcare clinic settings and systems and factors that they think will impact breast cancer follow-up care implementation. Additionally, the 12-item Organizational Readiness for Implementing Change measure will be used to examine change commitment and change readiness [86]. Leadership style will be measured by qualitative interviews and the Implementation Leadership Scale (ILS), a brief psychometrically strong measure that contains 12-items with four subscales of proactive, knowledgeable, supportive, and perseverant leadership [87]. Patient-clinician and healthcare team communication quality will be assessed through qualitative interviews asking patients, clinicians, and staff to assess their interactions.

### Patient-level outcome variables

The primary outcome variable will be breast cancer comprehensive follow-up care. This will be assessed by calculating the percentage of recommendations followed out of the total recommendations a given person is eligible to receive. Lower scores will indicate less comprehensive care. The number of recommendations may vary by patient depending on personal characteristics, like weight or smoking status. Research staff will collect data from the Electronic Health Record (EHR), conducting chart abstractions on 20 randomly selected patient records per practice at each time point. Measures of care coordination/management will be assessed from chart documentation of referral and/or treatment and screening for late and long-term effects, lifestyle, surveillance, and screening activities.

### Data analysis and learning evaluation feedback

#### Qualitative analysis

Ongoing analyses of data collected by our evaluation team will be fed back to the intervention team to inform them of progress, areas in need of contextual alignment, and opportunities for further adaptation. A working summary of emergent findings will be maintained and continuously updated as incoming data are added to the project. As a validity check of qualitative results, we will check relevant data interpretations against all new data using a constant comparison approach. We will note similarities and differences between practice sites and between successive adaptations to the intervention based on adaptations from feedback cycles as conveyed by practice members and patients with a history of breast cancer.

Intervention baseline, 6 month and 12 month quantitative and qualitative results will be summarized in brief reports with recommendations to be shared with IAC members for reflections on any changes needed. These analyses represent ongoing monitoring and feedback to inform optimal adoption of cancer follow-up care recommendations with real-time results influencing efforts to adapt recommendations to better fit local needs and contexts.

#### Quantitative analysis

The primary outcome for each patient will be the percent of recommendations followed out of those for which the patient was eligible. Descriptive statistics and visualization techniques will summarize the practice averages of these percentages, overall and by treatment group and time. Additionally, we will calculate the practice rates of providing each individual service from patients for whom the service was appropriate and compare these across treatment arms and times. In formal analyses, hierarchical linear models we will study the effect of treatment on the primary outcome. Specifically, the model will include a random effect for practice to account for intra-practice correlation between patients, effects for time (baseline/follow-up) and intervention group (intervention/control), and patient characteristics such as age, race, count of co-morbidities. To test whether the intervention affects changes in the outcome over time, we will use an F-test of the interaction between treatment arm and time. In exploratory analyses, hierarchical logistic models will examine effects of the intervention on changes over time on rates of fulfillment of individual services.

Since practices in this study will be chosen for their diversity in patient population and management systems, analyses will also be conducted separately for each practice. These analyses will allow us to qualitatively examine characteristics of practices in which the intervention was effective versus those where it was not.

##### Power calculations

For each individual patient, the percent of applicable recommendations that were followed will be calculated. Previous studies have seen rates between approximately 30% and 70% of individual recommendations being followed (e.g., colorectal cancer screening). Hence, we assume that the range for a vast majority of possible percentages lie between 20 and 80%, giving approximately a standard deviation of 15% (based on the Empirical Rule at with 95% of all observations falling within four standard deviations). Assuming this standard deviation, an interclass correlation of 0.1 [88], a dropout rate for practices of approximately 15%, and a mean difference in percentages between control and intervention practices of 15%, we would require 26 practices to achieve 90% power when conducting the test at the 0.05 level accounting for clustering within practice. If the interclass correlation was higher, say at 0.3, we would need to see a difference of 13% to achieve the same power with 26 practices.

## Discussion

This study is innovative in several key ways. First, its comprehensive focus on primary care delivery to patients with a history of breast cancer addresses the lack of care continuity for these patients. While limited studies have evaluated the impact of SCPs [40] and models of care for integrating primary care into breast cancer follow-up [89], we are not aware of any systematic studies that have used implementation science theoretical frameworks to holistically understand the inner (primary care) and outer (oncology and broader cancer policy) contextual factors that impact implementation of breast cancer care. Second, we use an innovative multi-level approach that combines two established frameworks— Exploration, Planning, Implementation, and Sustainment (EPIS) and the Practice Change Model—to simultaneously explore health system, practice, clinician, and patient-level factors that impact implementation of care for patients with a history of breast cancer. Third, we simultaneously engage different and disparate stakeholders (national experts and local implementers from oncology, primary care, nursing, social work, and patients) using mixed methods to triangulate data and gain a comprehensive understanding of multiple perspectives on delivery of breast cancer care in primary care settings. While there is literature that addresses different stakeholders, particularly clinicians [90, 91] and patients [92, 93], there is a significant gap in research that incorporates other perspectives. Finally, multiple professional organizations advocate clinical recommendations and guidelines for primary care; however, this will be the first time where all of these will be considered simultaneously, prioritized, and synthesized into actionable plans for providing care to long-term breast cancer patients in primary care settings.

Study findings are poised to inform development of scalable, high impact intervention processes in primary care to enhance long-term follow-up care for patients with a history of breast cancer. If successful, next steps would include working with a national primary care practice-based research network to implement a national dissemination study. Actionable activities and processes identified could also be applied to development of organizational and care delivery interventions for follow-up care for other cancer sites.

### Supplementary Information

**Additional file 1.**

## Data Availability

The datasets generated and/or analyzed during the current study will not be publicly available due to privacy concerns.
